# Definitive radiotherapy for early stage glottic cancer: a quarter-century of single-institution experience with long-term outcomes and late events

**DOI:** 10.1007/s00405-026-10176-1

**Published:** 2026-03-20

**Authors:** Atsuto Katano, Hiroyuki Ueno, Masanari Minamitani, Yusuke Ito, Koji Yamamura, Kenya Kobayashi, Yuki Saito, Hideomi Yamashita

**Affiliations:** 1 Department of Radiology, The University of Tokyo Hospital, 7-3-1 Hongo, Bunkyo-ku, Tokyo 113-0033 Japan; 2 Department of Otolaryngology - Head and Neck Surgery, The University of Tokyo Hospital, 7-3-1 Hongo, Bunkyo-ku, Tokyo 113-0033 Japan

**Keywords:** Glottic neoplasms, Laryngeal neoplasms, Radiotherapy, Treatment outcome, Survival analysis, Carotid stenosis, Cerebrovascular disorders, Radiation injuries

## Abstract

**Background:**

Definitive radiotherapy is a standard organ-preserving treatment for early-stage glottic cancer; however, real-world data describing very late adverse events after long-term follow-up remain limited.

**Methods:**

We retrospectively analyzed consecutive patients with stage I–II glottic squamous cell carcinoma treated with definitive external beam radiotherapy at a single institution over a 25-year period. Endpoints included overall survival (OS), cancer-specific survival (CSS), disease-free survival (DFS), and local control (LC). Time-to-event outcomes were estimated using the Kaplan–Meier method. The cumulative incidence of grade ≥ 3 adverse events was estimated with death as a competing risk, with vascular events and laryngeal necrosis–related events analyzed separately.

**Results:**

A total of 257 patients were included (median age, 70 years; 93% male); 169 (66%) had stage I and 88 (34%) had stage II disease. Median follow-up was 61.2 months (range, 3.0–265.7). In the overall cohort, OS at 5, 10, and 15 years was 86.5%, 81.8%, and 73.8%, respectively. DFS at 5 years was 81.7% in stage I and 67.5% in stage II (*p* = 0.0409). LC at 5 years was 89.6% in stage I and 82.0% in stage II (*p* = 0.154). Ten grade ≥ 3 adverse events were recorded, with a median time to onset of 95.0 months. The cumulative incidence of any grade ≥ 3 adverse event was 1.9% at 5 years and 4.6% at 10 years. Vascular events occurred in 8 patients (5-year 1.1%; 10-year 3.8%), while grade ≥ 3 laryngeal events occurred in 2 patients (0.8% at both 5 and 10 years).

**Conclusions:**

In this quarter-century real-world cohort, definitive radiotherapy for stage I–II glottic cancer achieved durable long-term outcomes with a limited incidence of severe late adverse events. The long latency of events, particularly vascular complications, refers the importance of extended survivorship follow-up and vigilant monitoring for uncommon but clinically meaningful late toxicity.

## Introduction

Laryngeal cancer has shown a declining trend globally; however, glottic cancer remains a substantial contributor to the global disease burden [1, 2]. Owing to its propensity to cause hoarseness at an early stage, glottic cancer is often detected while still localized. For early-stage glottic cancer, recommended treatment options include definitive radiotherapy, transoral laser microsurgery, and partial laryngectomy [3, 4]. Definitive radiotherapy is a primary treatment strategy and can preserve laryngeal function and maintain voice quality.

Conventional definitive radiotherapy typically requires a relatively long overall treatment time of approximately 6–7 weeks. Over the past two decades, however, hypofractionated radiotherapy has been increasingly adopted in routine practice. In 2005, a prospective randomized trial in T1 glottic cancer showed that 2.25 Gy per fraction, delivered over a shorter overall treatment time, improved local control compared with 2 Gy fractions, without an apparent increase in adverse events [5]. In a large single-institution series, Gowda et al. reported favorable outcomes in 200 patients with T1 glottic squamous cell carcinoma treated with 50.0–52.5 Gy in 16 fractions, with a 5-year local control rate of 93%, cause-specific survival of 97%, and rare severe toxicity [6]. More recently, the randomized JCOG0701 trial demonstrated that hypofractionated radiotherapy (60–64.8 Gy in 25–27 fractions) achieved progression-free and overall survival outcomes comparable to conventional fractionation (66–70 Gy in 33–35 fractions), with a trend toward fewer late adverse events [7].

However, despite these favorable outcomes, the clinical relevance of late adverse events that may emerge years after treatment has become increasingly recognized. In particular, irradiation of the neck may be associated with vascular sequelae, including carotid artery stenosis, potentially translating into an increased long-term risk of cerebrovascular events. A systematic review and meta-analysis reported a substantial prevalence of carotid stenosis after head and neck radiotherapy and suggested routine ultrasound screening beginning one year after treatment [8–10]. In addition, rare but clinically meaningful laryngeal complications, such as chondroradionecrosis, can occur and may necessitate intensive management and, in some cases, airway intervention [11]. Nevertheless, in routine clinical practice, the ascertainment and documentation of late adverse events are often heterogeneous, and real-world estimates may therefore differ from those reported in clinical trials or selected institutional series (Figs. and ).

Accordingly, we conducted a retrospective real-world cohort study of patients with stage I–II glottic cancer treated with definitive radiotherapy at our institution over a 25-year period. The aims of the present study were to characterize long-term oncological outcomes and to quantify the cumulative incidence of clinically relevant adverse events during follow-up.

## Methods

This single-institution retrospective cohort study was conducted in accordance with institutional policies and applicable ethical standards, including the principles of the Declaration of Helsinki. Institutional review board approval was obtained from the Research Ethics Committee of the Faculty of Medicine, The University of Tokyo (approval No. 3372-9). We analyzed consecutive patients with early-stage glottic laryngeal cancer treated with definitive radiotherapy from January 2000 to October 2025. Eligible patients met all of the following criteria: (i) histologically confirmed squamous cell carcinoma of the glottic larynx; (ii) clinical stage I–II disease, staged according to the 8th edition of the American Joint Committee on Cancer staging manual; (iii) receipt of definitive-intent external beam radiotherapy; (iv) no prior treatment for the primary tumor (recurrent cases were excluded); and (v) a minimum follow-up of 3 months.

All patients received definitive external beam radiotherapy using three-dimensional conformal radiotherapy with 4-MV photon beams. In accordance with our institutional protocol, patients were immobilized with a thermoplastic mask and underwent planning computed tomography [12]. Parallel-opposed lateral fields were used with a rectangular or conformal field size of 5 × 5 cm to 6 × 6 cm. Field borders were defined as follows: the superior border at the upper margin of the thyroid cartilage plus 5 mm; the inferior border at the lower margin of the cricoid cartilage; the anterior border set adequately anterior to the skin surface; and the posterior border positioned anterior to the vertebral body. Stage I disease was typically treated with either conventional fractionation (66 Gy in 33 fractions) or hypofractionation (60 Gy in 25 fractions), whereas stage II disease received either conventional fractionation (70 Gy in 35 fractions) or hypofractionation (64.8 Gy in 27 fractions). In a subset of patients with T2 disease treated before February 2010, concurrent chemotherapy was delivered in combination with conventional fractionation. After completion of radiotherapy, patients were followed according to institutional protocols. Follow-up typically included history and physical examination, flexible laryngoscopy, and imaging studies when clinically indicated.

All endpoints were measured from the start of radiotherapy. Overall survival (OS) was defined as time to death from any cause, cancer-specific survival (CSS) as time to death from glottic cancer, local control (LC) as time to first local recurrence at the primary site, and disease-free survival (DFS) as time to first recurrence or death from any cause, whichever occurred first. Adverse events were graded, when assessable, according to the Common Terminology Criteria for Adverse Events (CTCAE), version 5.0.

Time-to-event outcomes were estimated using the Kaplan–Meier method. Survival curves were generated for OS, CSS, DFS, and LC, with time measured in months. Patients without events were censored at the date of last follow-up. The cumulative incidence of grade ≥ 3 adverse events was estimated using the cumulative incidence function with death treated as a competing risk. Vascular events and laryngeal necrosis–related events were analyzed separately. Time was measured from the start of radiotherapy to the first event, with censoring at last follow-up. Baseline characteristics and treatment details were summarized using descriptive statistics. Continuous variables are presented as medians with interquartile ranges (IQRs) and ranges, and categorical variables as counts and percentages. All analyses were performed using R (version 4.1.2).

## Results

A total of 257 patients were included (Table 1). The median age was 70 years (range, 42–87), and 240 (93%) were male. Eastern Cooperative Oncology Group performance status was 0 in 233 (91%), 1 in 20 (8%), and 2 in 4 (2%). Clinical stage was I in 169 (66%) and II in 88 (34%). Concurrent chemotherapy was omitted in 227 (88%); 27 (11%) received low-dose cisplatin and 3 (1%) received low-dose nedaplatin. Radiotherapy was delivered as 60 Gy in 25 fractions (*n* = 81, 32%), 64.8 Gy in 27 fractions (*n* = 29, 11%), 66 Gy in 33 fractions (*n* = 74, 29%), or 70 Gy in 35 fractions (*n* = 73, 28%).

**Table 1:** Patient and treatment characteristics of the definitive radiotherapy cohort for glottic cancer

Variables	Number (Percentage)
Age: Median [Range]	70 [42–87]
Sex	
Male	240 (93%)
Female	17 (7%)
ECOG Performance Status	
0	233 (91%)
1	20 (8%)
2	4 (2%)
Clinical stage	
I	169 (66%)
II	88 (34%)
Concurrent Chemotherapy	
No	227 (88%)
Low-dose cisplatin	27 (11%)
Low-dose nedaplatin	3 (1%)
Dose and Fractionation	
60 Gy/25 fractions	81 (32%)
64.8 Gy/27 fractions	29 (11%)
66 Gy/33 fractions	74 (29%)
70 Gy/35 fractions	73 (28%)

**Fig. 1:**
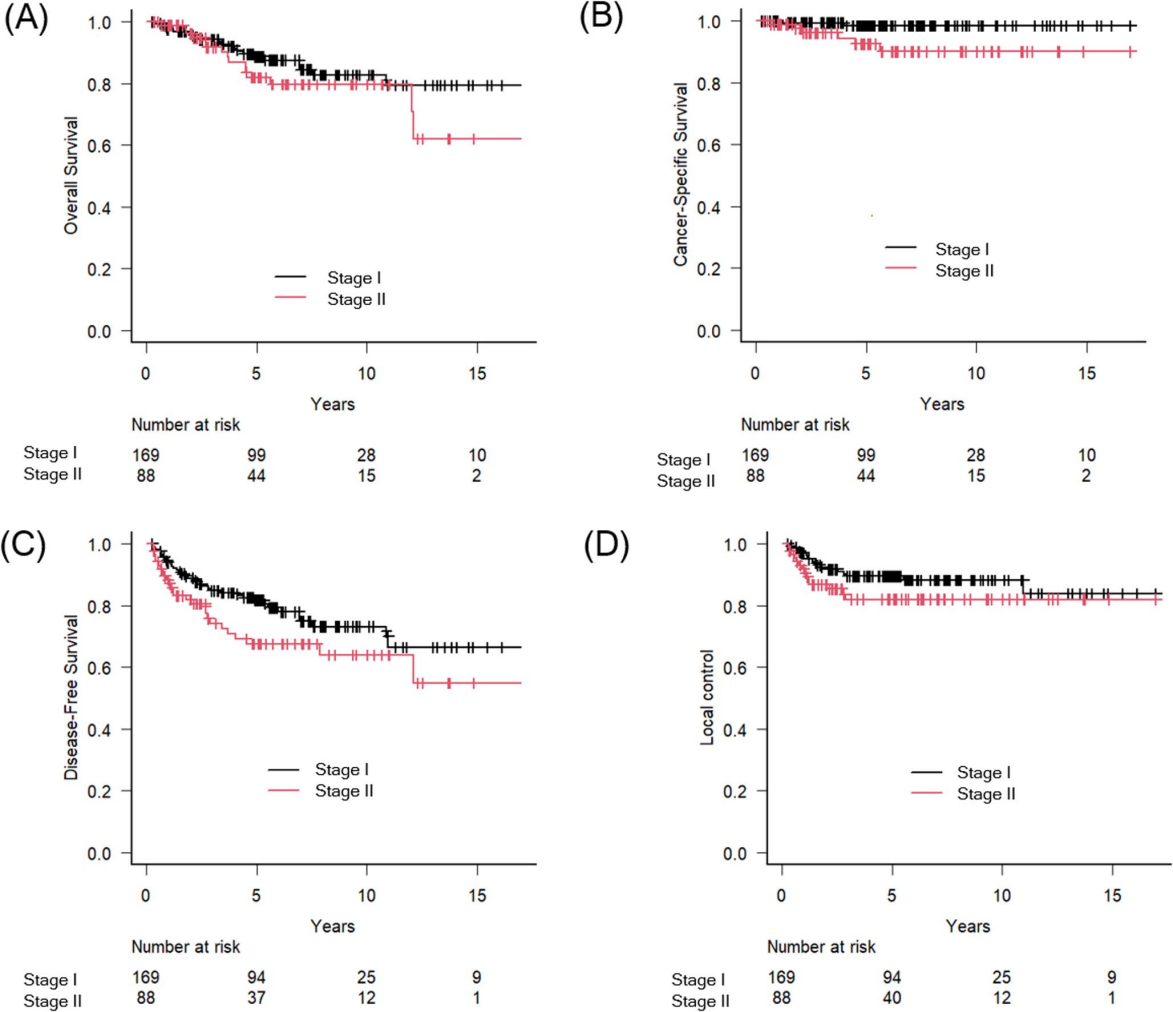
Kaplan–Meier curves of (**A**) overall survival, (**B**) cancer-specific survival, (**C**) disease-free survival, and (**D**) local control stratified by clinical stage (Stage I vs. Stage II) in the definitive radiotherapy cohort for glottic cancer

Median follow-up was 61.2 months (range, 3.0–265.7). In the overall cohort, OS at 5, 10, and 15 years was 86.5% (95% confidence interval [CI], 81.0–90.5%), 81.8% (74.8–87.0%), and 73.8% (62.0–82.5%), respectively (Fig. 1A). By clinical stage, OS at 5 and 10 years was 88.7% (82.2–93.0%) and 82.7% (73.6–88.9%) in stage I, and 81.9% (70.2–89.4%) and 79.7% (67.3–87.8%) in stage II, respectively. Among 39 deaths, 8 (20.5%) were attributable to glottic cancer, 14 (35.9%) to other malignancies, 12 (30.8%) to non-cancer causes, and 5 (12.8%) were of unknown cause. Disease-specific survival at 5 and 10 years was 98.5% (95% CI, 94.0–99.6%) and 98.5% (94.0–99.6%) in stage I, and 92.6% (82.8–96.9%) and 90.1% (78.6–95.6%) in stage II, respectively (Fig. 1B).

Disease-free survival at 5 years was 81.7% (74.6–87.1%) in stage I and 67.5% (55.2–77.1%) in stage II (*p* = 0.0409) (Fig. 1C). Recurrence was documented in 40 patients, most commonly at the primary site (*n* = 32), followed by regional lymph nodes (*n* = 6) and distant metastases (*n* = 2; lung, *n* = 1; mediastinal lymph nodes, *n* = 1). Local control at 5 years was 89.6% (83.5–93.5%) in stage I and 82.0% (71.2–89.0%) in stage II (*p* = 0.154) (Fig. 1D). Salvage treatments varied. Of 32 patients with primary-site recurrence, 29 underwent surgical salvage (total laryngectomy, *n* = 16; partial surgery, *n* = 13), whereas the remaining 3 received best supportive care, re-irradiation, or systemic therapy. For nodal recurrence (*n* = 6), neck dissection was performed in 4 patients and systemic therapy in 2. Both patients with distant metastases received cetuximab-containing systemic therapy.

**Fig. 2:**
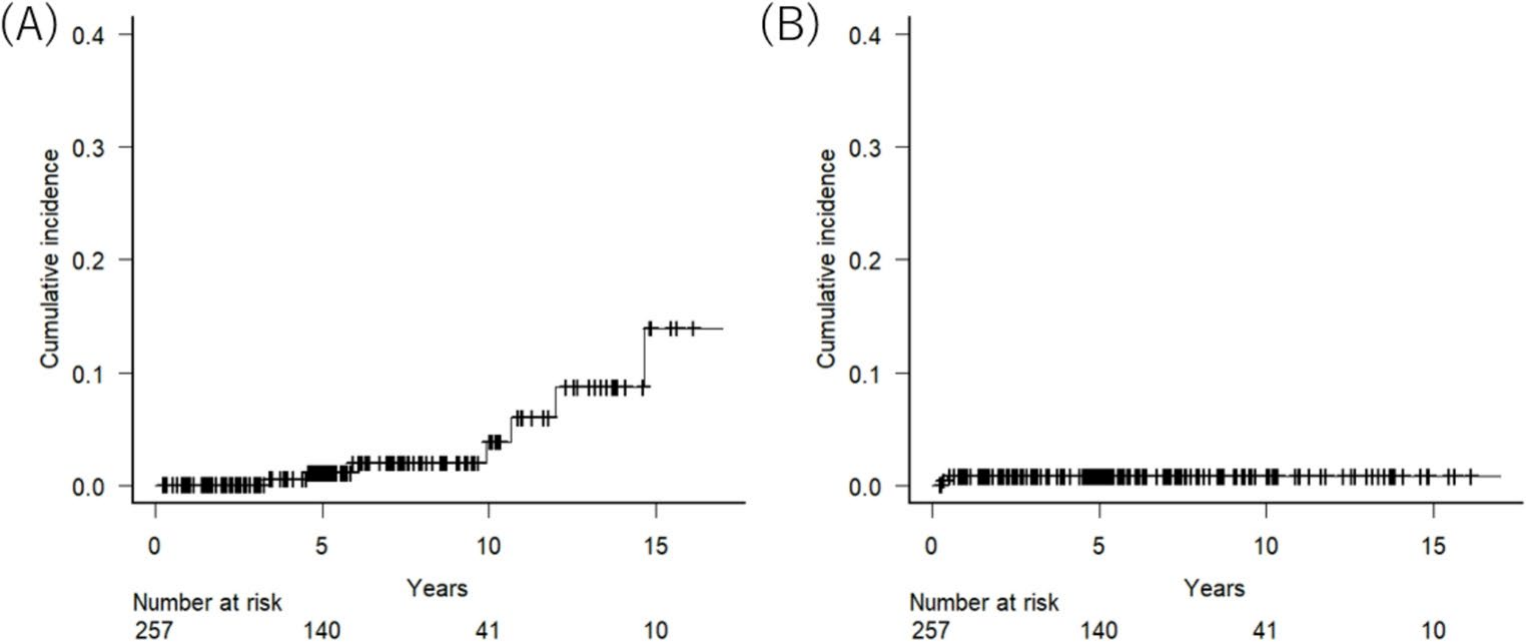
Cumulative incidence of late grade ≥ 3 adverse events after definitive radiotherapy for early-stage glottic cancer, specifically vascular events (**A**) and laryngeal related events (**B**)

A total of 10 grade ≥ 3 adverse events were recorded (Table 2), with a median time to onset of 95.0 months (range, 3.3–207.4). The cumulative incidence of any grade ≥ 3 adverse event was 1.9% (95% CI, 0.6–4.4%) at 5 years and 4.6% (1.5–10.5%) at 10 years. Eight patients experienced vascular events (cerebral infarction, *n* = 6; intracranial hemorrhage, *n* = 1; carotid artery stenosis, *n* = 1), corresponding to cumulative incidences of 1.1% (0.2–3.5%) at 5 years and 3.8% (1.0–9.9%) at 10 years (Fig. 2A). Two patients developed grade ≥ 3 laryngeal events (laryngeal stenosis, *n* = 1; chondronecrosis, *n* = 1), with cumulative incidences of 0.8% (0.2–2.6%) at both 5 and 10 years (Fig. 2B).

**Table 2:** Details of grade ≥ 3 adverse events and their management

Adverse event	Age	Sex	KPS	Clinical stage	Dose fractionation	Time to event (months)	Treatment	Smoking	Medical history
Stroke, grade 3 (cerebral infarction)	57	Male	90	1	66 Gy/33 fractions	70.9	Improved with inpatient treatment	10 cigarettes/day for 40 years	Chronic hepatitis C
Stroke, grade 3 (cerebral infarction)	64	Male	100	1	66 Gy/33 fractions	207.4	Improved with inpatient treatment	75 cigarettes/day for 30 years	Rectal cancer; diabetes; hypertension
Stroke, grade 3 (cerebral infarction)	67	Male	90	1	66 Gy/33 fractions	39.1	Improved with inpatient treatment	25 cigarettes/day for 47 years	Hypertension; diabetes; angina; hyperuricemia; gastroesophageal reflux disease
Stroke, grade 4 (cerebral infarction)	77	Male	90	2	70 Gy/35 fractions	119.1	Residual dysphagia and gait impairment after stroke onset	20 cigarettes/day for 55 years	Appendicitis; gallstones; hypertension; diabetes; Parkinson’s disease; vertebral compression fracture
Stroke, grade 3 (cerebral infarction)	67	Male	90	2	70 Gy/35 fractions	127.8	Improved with inpatient treatment	30 cigarettes/day for 40 years	HIV infection; acute pancreatitis; arrhythmia; hypertension; dyslipidemia; diabetes
Stroke, grade 3 (cerebral infarction)	70	Male	100	1	60 Gy/25 fractions	54.9	Improved with inpatient treatment	40 cigarettes/day for 18 years	Hemorrhoids; appendicitis (surgery); sudden hearing loss; bilateral femoral head osteonecrosis
Stroke, grade 3 (Intracranial hemorrhage)	65	Male	90	2	70 Gy/35 fractions	144.1	Improved with inpatient treatment	60 cigarettes/day for 40 years	Hypertension
Vascular disorder, grade 3 (carotid artery stenosis)	63	Male	100	1	66 Gy/33 fractions	175.8	Carotid endarterectomy with patch angioplasty	Former smoker (details unavailable)	Diabetes; dyslipidemia; diabetic kidney disease; hypertension
Laryngeal stenosis, grade 3 (laryngeal necrosis)	86	Female	80	2	64.8 Gy/27 fractions	5.4	Hospitalization and tracheostomy	Never smoker	Depression
Laryngeal stenosis, grade 3 (laryngeal chondronecrosis)	77	Male	90	1	60 Gy/25 fractions	3.3	Hospitalization and tracheostomy	15 cigarettes/day for 57 years	Rheumatoid arthritis

## Discussion

The present study adds single-institution real-world evidence on definitive radiotherapy for stage I–II glottic cancer. In our cohort, oncologic outcomes were durable over extended follow-up, and the incidence of clinically documented late adverse events was limited. These findings are broadly comparable to those reported in prior institutional series. Khan et al. reported a 20-year Cleveland Clinic experience with definitive radiotherapy for early (T1–T2) glottic squamous cell carcinoma, with 5-year local control rates of 94% (T1a), 83% (T1b), 87% (T2a), and 65% (T2b), and corresponding 10-year local control rates of 89%, 83%, 87%, and 53%, respectively [13]. They also reported a 5-year CSS of 95.9% and a 5-year OS of 76.8%; 73% of patients experienced significant voice improvement, while late events included chronic laryngeal edema (18%) and dysphagia (7%).

A central implication of this real-world cohort is that late events may emerge well beyond the conventional follow-up horizon used in many clinical studies. In our dataset, adverse events were uncommon overall; however, the median time to first recorded event was long (95.0 months), indicating that clinically meaningful complications may present many years after treatment. Texakalidis et al. conducted a meta-analysis of 19 studies and reported the prevalence, incidence, and severity of post-irradiation carotid stenosis in patients with prior head and neck irradiation [14]. Their findings suggest that vascular risk increases over time after radiotherapy, supporting the rationale for post-treatment surveillance.

Carotid-sparing intensity modulated radiation therapy (CS-IMRT) represents a technique-level strategy that may reduce carotid dose while maintaining target coverage. Feasibility studies have shown that CS-IMRT can reduce carotid exposure compared with conventional opposed lateral fields [15, 16]. The effectiveness of CS-IMRT is likely to depend not only on planning capability but also on reproducible treatment delivery and quality assurance. Rock et al. reported that, in a single-institution cohort of T2N0 glottic cancer treated with partial laryngeal IMRT, local control appeared to improve after the program transitioned to daily laryngeal soft-tissue matching image guided radiation therapy (IGRT) and a more accelerated fractionation approach [17]. In their analysis, multivariable analyses suggested that the IGRT matching strategy was associated with a lower risk of local failure, although the independent contribution of each programmatic change warrants further confirmation.

SBRT is a highly conformal treatment approach that can increase dose concentration within the target while reducing unnecessary irradiation of surrounding normal tissues [18, 19]. Gundog and Kiraz reported a retrospective study of 22 patients with early glottic cancer treated with SBRT (35–42.5 Gy in 5 fractions to the involved vocal cord), demonstrating favorable outcomes, with 5-year local control and larynx preservation rates of 94.7% and 89.7%, respectively, at a median follow-up of 59 months [20]. Sanguineti et al. also reported a prospective phase I/II study of SBRT (36 Gy in 3 fractions) for T1 glottic cancer in 33 patients, with 100% local control at 4 years and a median follow-up of 51.5 months [21]. However, accrual was discontinued because of concerns regarding late toxicity: 6 patients (18.2%) developed necrosis at a median of 14.9 months. These findings highlight both the promise of SBRT and the need for careful attention to late laryngeal toxicity.

A number of limitations need to be noted regarding the present study. First, as this was a real-world retrospective cohort with a long observation period, treatment techniques and dose–fractionation schedules were not fully standardised. Second, adverse events were identified from routine clinical records rather than protocol-based assessments; therefore, some events may have been missed, misclassified, or incompletely graded. Third, because pre-RT endoscopic and imaging documentation was not uniformly available, we could not consistently determine whether a visible lesion persisted at the initiation of radiotherapy. Finally, given the retrospective design, causality between radiotherapy and vascular events cannot be established, particularly in patients with baseline vascular risk factors.

Despite these limitations, this study provides long-term real-world outcome data that are useful for daily clinical practice. Our findings support definitive radiotherapy as a reliable organ-preserving treatment for early-stage glottic cancer. They also highlight the importance of long-term follow-up that remains alert to uncommon but clinically important late events. Future research should focus on identifying patients at higher risk of late vascular and laryngeal complications.
